# Portable Vertical Graphene@Au-Based Electrochemical Aptasensing Platform for Point-of-Care Testing of Tau Protein in the Blood

**DOI:** 10.3390/bios12080564

**Published:** 2022-07-25

**Authors:** Yibiao Liu, Xingyun Liu, Mifang Li, Qiong Liu, Tailin Xu

**Affiliations:** 1 Longgang District Central Hospital of Shenzhen, Shenzhen 518116, China; liuyibiao12345@126.com (Y.L.); limifang01@163.com (M.L.); 2 School of Biomedical Engineering, Health Science Center, College of Life Sciences and Oceanography, Shenzhen University, Shenzhen 518060, China; liuxingyunfairy20@163.com

**Keywords:** portable electrochemical aptasensor, Alzheimer’s disease, tau protein, vertical graphene

## Abstract

Alzheimer’s disease (AD) is a long-term neurodegenerative disease that poses a serious threat to human life and health. It is very important to develop a portable quantitative device for AD diagnosis and personal healthcare. Herein, we develop a portable electrochemical sensing platform for the point-of-care detection of AD biomarkers in the blood. Such a portable platform integrates nanoAu-modified vertical graphene (VG@Au) into a working electrode, which can significantly improve sensitivity and reduce detection limit due to the large specific surface, excellent electrical conductivity, high stability, and good biocompatibility. The tau protein, as an important factor in the course of AD, is selected as a key AD biomarker. The results show that the linear range of this sensing platform is 0.1 pg/mL to 1 ng/mL, with a detection limit of 0.034 pg/mL (S/N = 3), indicating that this portable sensing platform meets the demand for the detection of the tau protein in the blood. This work offers great potential for AD diagnosis and personal healthcare.

## 1. Introduction

Alzheimer’s disease (AD), one of the most prevalent neurodegenerative diseases, poses a serious threat to human life and health and is rapidly becoming one of the most expensive, lethal diseases in this century [1]. Until now, there is still no effective treatment for AD. Early diagnosis, early detection, and early intervention are a significant and effective strategy [1,2]. The tau protein, as a defining characteristic of AD, is recognized as an important biomarker of AD [3,4]. The quantitative determination of the tau protein in cerebrospinal fluid (CSF) is the gold standard for the diagnosis of AD. However, the AD diagnosis based on CSF is limited to the trouble of obtaining CSF. The detection of the tau protein in easily accessible blood is a possible alternative method [1,5,6]. However, the concentration of tau protein in the blood is extremely low (~pg/mL and lies beyond the detection range of the classic enzyme-linked immunosorbent assay (ELISA). Therefore, it is essential to develop an ultrasensitive, noninvasive, and portable quantitative device for detecting tau protein in the blood.

So far, many detection methods have been developed for the detection of the tau protein, including surface plasmon resonance (SPR) [7,8], surface-enhanced Raman spectroscopy (SERS) [9], field-effect transistors [10,11], colorimetry [12], fluorescence [13,14], and electrochemistry [15,16,17]. Among these methods, a portable electrochemical biosensor has tremendous potential for home health monitoring and personal healthcare due to its easy miniaturization, high sensitivity, and low cost [18,19,20,21,22]. A portable biosensor can be particularly significant as home health monitoring and personal healthcare become more and more common during the COVID-19 pandemic [23,24]. In recent years, portable sensors have been widely used in the field of disease diagnosis [25,26,27], e.g., a commercial, portable blood glucometer for detecting blood glucose, portable smartphone-based devices for monitoring cardiovascular diseases [28], portable biosensors for coronavirus disease [29], and portable electrochemical biosensors for the early diagnosis of periodontal disease [30]. However, very few portable electrochemical sensing platforms are reported in the field of AD diagnosis [16,31].

**Figure 1:**
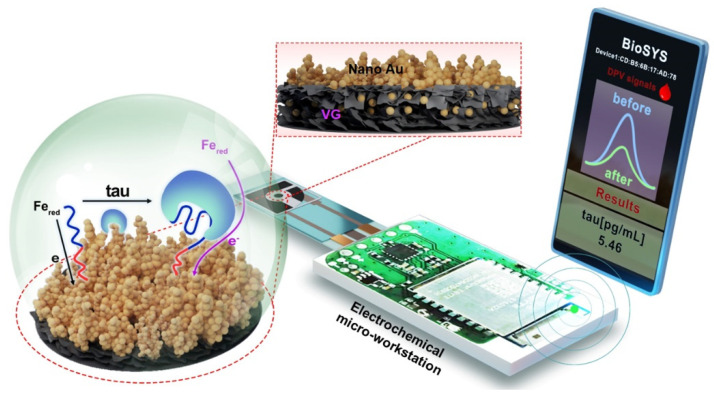
Schematic illustration of electrochemical aptasensing platform based on VG@Au for detecting tau protein.

In this work, a portable electrochemical aptasensing platform is developed for the point-of-care detection of tau protein in the blood. In this electrochemical sensing system, vertical graphene (VG) modified with nanoAu (VG@Au) is used as an electrode material because of its large specific surface area, excellent electrical conductivity, high carrier mobility, good chemical stability, and outstanding biocompatibility [32,33], which improves sensitivity and reduces detection limit. In addition, nanoAu is also used to bind with aptamer. As shown in Figure 1, the entire electrochemical aptasensing system consists of an electrochemical micro-workstation (control system, size: 2 cm × 1 cm × 0.5 cm), a paper-based three-electrode sensing system (size: 2 cm × 0.7 cm × 0.1 cm), and a smartphone (results output system). The electrochemical micro-workstation is used for signal control, which connects to the smartphone by Bluetooth. The result is shown in BioSYS APP. The internal structure of the electrochemical micro-workstation is displayed in Figure 1, and the detail is shown in our previous paper [16]. The DNA aptamer of the tau protein was immobilized on the surface of VG@Au by Au-S. When the tau protein binds with aptamer, the spatial structure of DNA aptamer changes, hindering the electron transfer on the electrode surface. During this process, the peak current of the differential pulse voltammetry (DPV) is reduced. The concentration of the tau protein could be determined by measuring the DPV peak current changes. This sensing platform exhibits a low detection limit of 0.034 pg/mL and a wide linear range of 0.1 pg/mL–1.0 ng/mL. This portable VG@Au-based electrochemical sensing platform shows tremendous potential for AD diagnosis at home and in personal healthcare.

## 2. Materials and Methods

### 2.1. Chemicals and Reagents

Tau441 was purchased from Abcam Ltd. and stored at −20 °C. The DNA aptamer of tau protein (5′-SH-(CH2)6-CAGCACCGTCAACTGAATGGGTTGGCCGGGCAGCGGGGGGTAGGCTGGT GATGCGATGGAGATGT-3′) was synthesized by Sangon Biotech Co., Ltd. (Shanghai, China) according to the reference [34], and stored at 4 °C. Human serum albumin (HSA), glucose (GLU), β-amyloid peptide (Aβ), potassium chloride (KCl), ascorbic acid (AA), potassium ferricyanide/ferrocyanide (K_3_[Fe(CN)_6_]/K_4_[Fe(CN)_6_]), mercaptoethylamine (MCH), and PBS buffer (pH = 7.4, 10 mM) were obtained from Sigma-Aldrich (Shanghai, China). All chemicals were of analytical grade and used directly. Ultrapure water (18.2 MΩ·cm) was used in all experiments.

### 2.2. Apparatus

The morphology of VG and VG@Au was characterized by scanning electron microscopy (SEM, ThermoFisher, FEI Apreo S, Waltham, MA, USA). The cross-section view of VG@Au and elemental mapping analysis were characterized using high-resolution scanning electron microscopy (SEM, ThermoFisher, FEI Apreo S, Waltham, MA, USA). All the electrochemical measurements were performed by an electrochemical micro-workstation that was customized from Refresh AI Biosensor Co., Ltd, Shenzhen, China.

### 2.3. Preparation of VG@Au Electrode

The VG was prepared by chemical vapor deposition, and the paper-based VG electrode was customized from ShenzhenYickxin Technology R&D Co., Ltd, Shenzhen, China. Then, the nanoAu was modified on the surface of the VG by electrodeposition. The electrodeposition was processed in H_2_SO_4_ (0.5 M) containing 10 mM HAuCl_4_ at −1.8 V. The deposition time was 80 s.

### 2.4. Fabrication and Analytical Performace of Portable Sensing Platform

Firstly, the VG@Au electrode surface was rinsed with ultrapure water before modification, and then dried at room temperature. Secondly, the DNA aptamer of the tau protein (10 μM) was dropped onto the VG@Au electrode surface and incubated for 1 h at 37 °C. Then, mercaptoethylamine (MCH, 1 mM) was used to block non-specific binding sites. Finally, the 10 μL tau protein prepared with PBS buffer was dripped onto the electrode surface and incubated for 1 h at 37 °C. After each step, the VG@Au electrode surface was rinsed three times with PBS buffer.

The determination of the tau protein was carried out by differential pulse voltammetry (DPV) using a three-electrode system. (The VG@Au electrode was used as the working electrode. An Ag/AgCl electrode was the reference electrode, and another VG electrode was used as the counter electrode.) The electrochemical assay was implemented by differential pulse voltammetry (DPV) in 5 mM K_3_(Fe(CN)_6_)/K_4_(Fe(CN)_6_) solution containing 0.1 M KCl. The scan range was taken from −0.2 V to 0.4 V with a scan rate of 0.10 V/s. When the different concentration of the tau protein bonded with the aptamer, the changing value of the peak current (ΔI) was recorded successively, which was used to calculate the concentration of the tau protein. The selectivity of this portable VG@Au-based electrochemical sensing platform was investigated by DPV in PBS buffer containing BSA, Aβ protein, and tau protein.

### 2.5. Detection of Clinical Serum Samples

Simply, DNA aptamer (10 mM) was dropped onto the surface of VG@Au and incubated for 1 h. Then, 20 μL sample (4 μL samples diluted with 16 μL PBS buffer) was added to the VG@Au electrode surface and incubated for 1 h at 37 °C. During this process, the peak current changing value of DPV signals was recorded.

## 3. Results and Discussion

### 3.1. Preparation and Characterization of VG@Au

**Figure 2:**
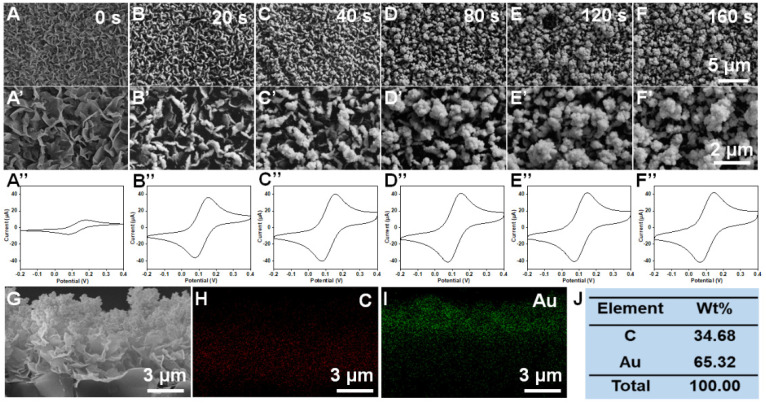
Effect of deposition time on the morphology and electrochemical performance of VG@Au. SEM images of VGs modified with nano Au at different deposition time (**A**,**A’**) 0 s; (**B**,**B’**) 20 s; (**C**,**C’**) 40 s; (**D**,**D’**) 80 s; (**E**,**E’**) 120 s; (**F**,**F’**) 160 s. (**A’’**–**F’’**) are the corresponding CV curves of figure (**A**–**F**). The cross-section view morphology (**G**) and corresponding element distribution characterization (**H**–**J**) of VG@Au at deposition time of 80 s.

The VG was prepared by chemical vapor deposition, and the surface morphology is shown in Figure 2A,A’. Many lamellar structures were observed. The peak current of CV was about 8.9 μA, which demonstrates that VG has good electrical conduct performance. To further improve the electrical conductivity and electron transfer rate, the VG surface was modified with nanoAu by electrodeposition. The nanoAu content of the VG@Au electrode was optimized by regulating deposition time. As shown in Figure 2B,B’, when the deposition time was 20 s, some gold particles appeared on the surface of the graphene. As deposition time increased, a nanoflower structure consisting of many gold particles was observed (Figure 2C–F and 2C’–F’). According to the CVs in Figure 2A’’–F’’, the peak current increased with increasing deposition time. When the deposition time was 80 s, the peak current increased greatly compared to the VG electrode, reaching 41.1 μA. However, the peak current changed little with a further increase in deposition time. Therefore, we selected 80 s as the optimized deposition time.

In addition, a cross-section view morphology of VG@Au is shown in Figure 2G. We found that the nanoAu was mainly deposited in the middle-upper part of the VG. To further observe the distribution of gold, an energy dispersive X-ray (EDX) was performed, and the element distribution of C and Au is shown in Figure 2H–J, demonstrating that nanoAu was displayed mainly in the middle-upper part of the VG.

### 3.2. Fabrication and Analytical Performance of Portable Electrochemical Sensing Platform

**Figure 3:**
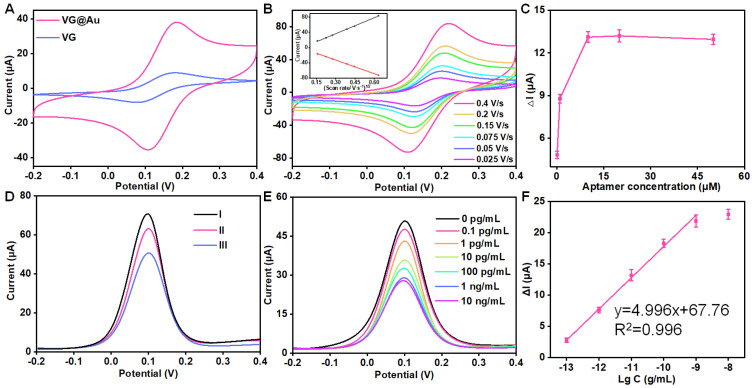
The analytical performance of the portable VG@Au-based electrochemical aptasensing platform for point-of-care detection of tau protein. (**A**) A electro-active area comparison (CVs) of VG and VG@Au electrode in 5 mM (Fe(CN)_6_)^3−^/(Fe(CN)_6_)^4−^ solution containing 0.1 M KCl at 0.01 V/s. (**B**) CVs of VG@Au electrode at different scan rates. The inset is the relationship between peak current and the square root of scan rate. (**C**)The optimization of DNA aptamer. (**D**) DPV signals of VG@Au (line I), VG@Au + aptamer (line II), VG@Au + aptamer + MCH (line III). (**E**) DPV signals of different concentration tau protein in 5 mM (Fe(CN)_6_)^3−^/(Fe(CN)_6_)^4−^ solution containing 0.1 M KCl at 0.01 V/s. (**F**) The corresponding calibration curve of figure (**E**).

The paper-based VG electrode was customized by Shenzhen Yickxin Technology R&D Co., Ltd, Shenzhen, China. Then, the VG electrode surface was modified with nanoAu by electrodeposition, and the electroactive area of VG@Au was evaluated. As shown in Figure 3A, the electroactive area of the VG@Au electrode was remarkably larger than that of the VG electrode and bare Au. Moreover, CVs at different scan rates were carried out, and the result showed that the peak current grew linearly as the square root of the scan rate increased, which indicated that it was a diffusion-limited process. (Figure 3B) The VG@Au-based electrochemical aptasensor was prepared by successive self-assembly steps. This process was assessed by DPV. Firstly, the DNA aptamer of the tau protein was immobilized on the electrode surface. After modifying the aptamer, the peak current of the DPV signal decreased, indicating that the aptamer was successfully modified on the VG@Au surface. (Figure 3D, line II) Secondly, MCH was used to block the nonspecific adsorption sites. (Figure 3D, line III) The peak current was further reduced. Finally, a different concentration of the tau protein was added to the electrode surface. During this period, the DPV signal was recorded. The quantification of the tau protein could be calculated by the changing value of the peak current.

To obtain better analytical performance, we optimized the concentration of aptamer. The result showed that the optimized concentration was 10 μM (Figure 3C). Under optimized conditions, the tau protein was quantitatively detected by the portable VG@Au electrochemical aptasensing platform according to the peak current variation of DPV signals (ΔI). The result indicated that the peak current was reduced, and ΔI increased as the tau protein concentration increased. The ΔI grew linearly as the logarithm of the tau protein concentration increased. (Figure 3E,F) The detection limit was 0.034 pg/mL. (S/N = 3), and the linear range was from 0.1 pg/mL to 1 ng/mL. The LOD was calculated by three times the standard deviation of the blank according to the reference. [29,30] Compared to previous reports, our developed portable electrochemical sensing platform exhibited excellent analytical performance. A comparison was shown in Table 1. This portable electrochemical aptasensing platform showed a lower LOD. The concentration of the tau protein is several picograms per milliliter in the blood. The detection limit of this sensor is 0.034 pg/mL, which meets the requirements for the detection of the tau protein in blood.

### 3.3. Selectivity and Stability

**Figure 4:**
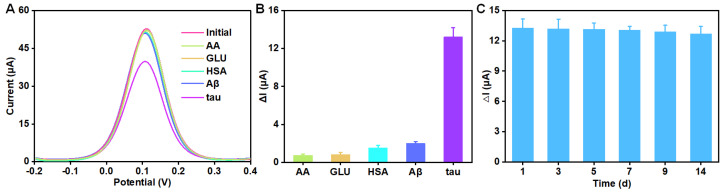
The selectivity and stability of this sensor. (**A**) DPV signals of 10 pg/mL tau and 10 ng/mL AA, GLU, HSA and Aβ. (**B**) A comparison of this aptasensor response towards tau and interfering molecules. (**C**) The changing value (ΔI) of peak current for detecting 10 pg/mL tau at different times.

The selectivity and stability were also investigated. As shown in Figure 4A,B, when 10 pg/mL tau protein was added, the DPV signal decreased significantly. However, when 1000-fold AA, GLU, HAS, or Aβ were added, there as little change in DPV signals, indicating that this sensing system had an outstanding selectivity. What is more, the stability of this electrochemical sensing platform based on VG@Au was evaluated by multiple tests of 10 pg/mL tau protein, and the result is shown in Figure 4C. After 14 days, the ΔI remained above 90% of its original level, which demonstrated that this portable electrochemical aptasensing platform based on VG@Au had excellent stability.

### 3.4. Application of Portable Electrochemcial Aptasensing Platform in Clinical Samples

To verify the clinical value of this portable VG@Au-based electrochemical aptasensing platform, we detected three clinical samples and compared the results with the results from agent of Quanterix Co., Ltd. (Billerica, MA, USA) in China, which is a professional AD blood-testing company. As shown in Table 2, there was no significant difference between our results and that of the company. These results demonstrated that our designed portable VG@Au-based electrochemical sensing platform could be applied to the detection of the tau protein in clinical samples. In addition, this portable electrochemical aptasensing platform could transfer signals to a smartphone by Bluetooth, and the result appeared on smartphone APP for the user to see.

## 4. Conclusions

In conclusion, a portable electrochemical aptasensing platform was developed for the point-of-care detection of tau protein in the blood. This portable platform consists of a VG@Au-based sensing system, an electrochemical micro-workstation, and a smartphone. The 3D structure of VG@Au exhibits a large specific surface, excellent electrical conductivity, high stability, and good biocompatibility, which significantly improved the sensitivity of the sensing system and reduced the detection limit. As a result, the detection limit of the portable VG@Au-based sensing platform was 0.034 pg/mL, which satisfies the demand for the detection of tau protein in the blood. The results of the test appeared on a smartphone APP for the user to see. This portable electrochemical sensing platform could be used for point-of-care tests of other biomarkers. In a follow-up work, we will use this sensing platform for detecting glucose and amyloid peptides. This work provides great potential for AD diagnosis in the home and for personal healthcare.

## Figures and Tables

**Table 1:** A comparison between this electrochemical aptasensor and other methods for the detection of tau protein.

Methods	Biomarkers	LOD	Linear Range	References
Electrochemistry	T-tau	0.142 pg/mL	0.1–1000 pg/mL	[16]
Electrochemistry	T-tau	0.059 pg/mL	0.1–100 pg/mL	[15]
Electrochemistry	tau	1.7 pg/mL	0–2.5 ng/mL	[35]
Electrochemistry	tau	0.46 pg/mL	4.6 pg/mL–4.6 μg/mL	[36]
Electrochemistry	Tau381	28 pg/mL	40–4000 pg/mL	[37]
FET sensors	tau	0.01 pg/mL	10 fg/mL–1ng/mL	[11]
FET sensors	tau	1.003 pg/mL	0.1pg/mL–100 ng/mL	[10]
Photoelectrochemistry	Tau381	0.013 pg/mL	0–40 ng/mL	[38]
Fluorescence	Tau441	0.56 pg/mL	0–46 pg/mL	[13]
LSPR	tau	46 ng/mL	23–575 ng/mL	[7]
Electrochemistry	tau	0.034 pg/mL	0.1 pg/mL–1 ng/mL	This work

**Table 2:** Determination of tau in clinical samples and a comparison between our sensor and Quanterix Co., Ltd.

Sample	Biomarkers	This Sensor (pg/mL)	Quanterix Co. (pg/mL)	Margin of Error
1	tau	2.19 ± 0.15	2.08 ± 0.08	+5.29%
2	tau	4.12 ± 0.11	3.93 ± 0.12	+4.83%
3	tau	4.28 ± 0.18	4.37 ± 0.09	−2.06%

## Data Availability

The data presented in this study are available on request from the corresponding author.
